# Adherence to Levothyroxine Tablet in Patients with Hypothyroidism

**DOI:** 10.7759/cureus.4624

**Published:** 2019-05-08

**Authors:** Rohan Kumar, Faizan Shaukat

**Affiliations:** 1 Medical Education and Simulation, Jinnah Sindh Medical University, Karachi, PAK; 2 Internal Medicine, Jinnah Postgraduate Medical Center, Karachi, PAK

**Keywords:** morisky medication adherence scale, hypothyroidism, levothyroxine, medication adherence, drug adherence

## Abstract

Introduction

Patients with hypothyroidism are managed with life-long levothyroxine (LT4) therapy. However, as with other chronic illnesses, drug adherence (DA) is a prominent issue in these patients. The aim of this study is to identify the extent of DA to LT4 in hypothyroidism patients and study the clinical factors contributing to DA in these patients.

Methods

This cross-sectional study assessed patient adherence to LT4 therapy by Modified Morisky Adherence Scale (MMAS). Factors predicting the pattern of medication adherence were also assessed in all patients. Data were entered and analyzed using SPSS v. 22.0.

Results

On MMAS, 79 (27.3%) participants indicated low adherence, 117 (40.48%) indicated medium adherence, and 93 (32.2%) participants indicated high adherence. Regular endocrinologist visits and knowledge about medication were highest in high adherent patients (p < 0.05). Need for assistance in taking medication, avoidance of medication with symptomatic relief and busy work schedule was highest in low adherent patients (p < 0.05).

Conclusion

Patients with hypothyroidism showed moderate adherence to their treatment.

## Introduction

Adherence (or compliance), in healthcare, is defined as the extent to which a patient is in conformity with the actual regimen prescribed by the healthcare provider. It encompasses overall behavior of the patients ranging from taking timely medicines and adjusting dietary habits to modifying their life-style [1]. In developing countries, given the scarcity of resources and inequities in healthcare system, non-adherence is somewhat comprehensible, but in developed countries only 50% adherence in patients suffering from chronic illnesses is quite alarming [2-3].

Although, it may be inferred that patients in clinical trials tend to behave ideally under scrutiny, leading to a false high rate of adherence; yet, only an average adherence rate is reported by clinical trials in patients with chronic diseases [4-5]. A high medical adherence rate helps reduce total healthcare costs by reducing the number of hospitalizations and emergency room visits for several common diseases [6]. Good drug adherence (DA) also leads to lower mortality rates as opposed to poor adherence [7].

A major factor contributing to adherence is ‘health literacy’ which is patients’ ability to obtain process and understand all the information related to the progress and the complications of their disease such that they are more adherent to a treatment regimen. Improving health literacy not only involves the patients in their treatment plan, it also helps them make informed decisions about their health. This is only possible with effective communication and discussions with the healthcare providers [8]. DA has been a global issue in patients with chronic diseases. Many studies have been conducted on major chronic conditions including hypertension [8], diabetes mellitus [9], heart failure [10], and epilepsy [11].

Hypothyroidism is a chronic illness with varying prevalence in different geographical locations and a higher gender predisposition towards women [12]. Patients with hypothyroidism are managed with life-long levothyroxine (LT4) therapy, with dose adjustment according to their circulating thyroid stimulating hormone levels [13]. Missed doses of LT4 result in symptom exacerbation and can even present as a neurocognitive emergency [14]. The factors associated with missed doses or noncompliance to LT4 may be patient-related, diseases-related, or even physician-related [15]. The aim of this study is to identify patients nonadherent to their LT4 therapy and study the clinical factors contributing to DA in these patients.

## Materials and methods

This observational, cross-sectional study was conducted in the thyroid clinic of outpatient medical department of a public hospital in Karachi from July - December 2018 after ethics approval. Patients, of age ≥18 years, who were attending the clinic with diagnosed hypothyroidism for at least one year, and could read and understand English, were included in the study after informed consent.

A self-administered performa was created which included patient gender, duration of LT4 prescription, clinical factors related to the patient, and Modified Morisky Adherence Scale (MMAS). MMAS is an 8-item relatively simple and practical tool for screening patient non-adherent to their medications in outpatient settings. It was first administered in patients with hypertension and has a reliability score of 0.83 and sensitivity of 93% [16]. The first seven items are to be responded with a “yes” or a “no.” “No” has one point and “yes” has zero points for all items. The eighth item is rated on five-point scale A-E. The response A scores one point, B scores 0.25, C scores 0.50, D scores 0.75, and E scores O points. Cumulative score of MMAS-8 is 0-8. Low adherence is indicated by MMAS <6, medium adherence is MMAS 6 - <8, and a score of 8 indicated high DA [17]. The clinical factors predicting pattern of DA included in this study were adapted from Shakya et al. [15].

Data were entered and analyzed using SPSS version 22 (NY:USA). Frequency and percentages were calculated for categorical data and mean and standard deviation (SD) was calculated for continuous variables. Internal consistency of the 8-item MMAS and 9-item clinical factors scale was calculated using Cronbach Alpha. Chi square was applied for comparison of categorical data. P value ≤0.05 was taken as significant.

## Results

Two hundred and eighty-nine (289) individuals with diagnosed hypothyroidism participated in this study. There were 201 (69.55%) women and 88 (30.44%) men. There were 140 (48.4%) participants who had been prescribed LT4 for more than 5 years, and 149 (51.55%) participants who had been prescribed LT4 for 1-5 years.

The internal consistency of MMAS in this study was 0.80 and that of the clinical factors scale was 0.75. On MMAS, 79 (27.3%) participants scored <6 and were indicated to have low DA, 117 (40.48%) scored 6 - <8 and were indicated to have medium DA, and 93 (32.2%) participants scored 9 indicating high DA. The distribution of extent of DA and its relationship with patient gender and duration of prescription are shown in Table 1.

**Table 1:** Relationship of level of drug adherence with patient gender and duration of LT4 prescription DA, drug adherence; LT4, levothyroxine

Level of drug adherence	Total sample (n=289)	Gender			Duration of LT4 prescription		
		Male (n=88)	Female (n=201)	P value	1-5 years (n=149)	> 5 years (n=140)	P value
High DA	93 (32.2%)	43 (48.8%)	50 (24.8%)	0.0002	28 (18.7%)	65 (46.4%)	<0.00001
Medium DA	117 (40.5%)	29 (32.9%)	88 (43.7%)		87 (58.3%)	30 (21.4%)	
Low DA	79 (27.3%)	16 (18.2%)	63 (31.3%)		34 (22.8%)	45 (32.1%)	

The patients’ DA levels were then correlated with clinical factors related to the disease. These are summarized in table 2. Among individuals with high DA patients, 72 (77.4%) visited their doctors regularly, 45 (48.3%) were educated about their disease and 68 (73.1%) about their medication, 55 (59.2%) could afford their medicines, medicines were accessible to 52 (55.9%), 25 (26.8%) needed assistance in taking medications and had busy work schedule each, 14 (15.1%) missed doses when symptoms alleviated, and 11 (11.8%) missed doses due to side effects. The responses on education about the disease and the medications, regular endocrinologist visits, medication affordability, work schedule, and need of assistance were significantly correlated with the level of DA as shown in Table 2.

**Table 2:** Impact of disease-related clinical factors on the extent of drug adherence DA, drug adherence

Clinical factors related to the disease	High DA (n=93)	Medium DA (n=117)	Low DA (n=79)	P value
Regularly visit endocrinologist				
Yes	72 (77.4%)	82 (70.1%)	25 (31.6%)	<0.00001
No	21 (22.5%)	35 (29.9%)	54 (68.3%)	
Knowledge given about the condition by the managing endocrinologist				
Yes	45 (48.3%)	72 (61.5%)	37 (46.8%)	0.06
No	48 (51.6%)	45 (38.4%)	42 (53.1%)	
Knowledge given about the medication by the managing endocrinologist				
Yes	68 (73.1%)	69 (58.9%)	24 (30.3%)	<0.00001
No	25 (26.8%)	48 (41.0%)	55 (69.6%)	
Need assistance taking medication				
Yes	25 (26.8%)	34 (29.1%)	48 (60.7%)	<0.00001
No	68 (73.1%)	83 (70.9%)	31 (39.2%)	
Medication affordability				
Yes	55 (59.2%)	84 (71.7%)	29 (36.7%)	<0.00001
No	38 (40.8%)	33 (28.2%)	50 (63.3%)	
Medication accessibility				
Yes	52 (55.9%)	67 (57.2%)	35 (44.3%)	0.16
No	41 (44.1%)	50 (42.7%)	44 (55.7%)	
Busy work schedule				
Yes	25 (26.8%)	53 (45.3%)	46 (58.2%)	0.0001
No	68 (73.1%)	64 (54.7%)	33 (41.7%)	
Avoid medication when symptomatic relief				
Yes	14 (15.1%)	41 (35.1%)	60 (75.9%)	<0.00001
No	79 (84.9%)	76 (64.9%)	19 (24.1%)	
Avoid medication due to side affects				
Yes	11 (11.8%)	19 (16.2%)	20 (25.3%)	0.06
No	82 (88.2%)	98 (83.7%)	59 (74.7%)	

## Discussion

More than two-third individuals with hypothyroidism are medium-to-high adherent to their prescriptions. More men were high adherent and more women were moderate-to-low adherent. Patients with 1-5 years of LT4 prescription were moderately adherent and patients with longer duration of prescription showed high adherence. It was seen that patient education about the disease and the medications, regular visits to the endocrinologist, medication affordability, work schedule, and assisted medication taking were significantly correlated with the level of DA. Medication side effects and accessibility was not significantly correlated to extent of DA.

To the best of our knowledge, this study is primary in reporting the extent of medication compliance among hypothyroid patients in Pakistan. Although, this is a single-center study and cannot be generalized to the situation prevailing in the country, still, it provides a very concrete picture as this hospital is the largest public tertiary care in the entire province. An important limitation to this study is that the DA rate was self-reported and not checked or confirmed via number of empty pill leaflets, drug card, or any other method.

In an observational cross-sectional study, conducted in 320 patients in north-eastern Italy [18], the study population had 1.9% low adherers (compared to 27.3% in our study), 10.9% medium adherers (compared to 40.5% in ours), and 87.2% high adherers (as opposed to 32.2% in this study). However, this study also showed a higher adherence rate in patients taking liquid LT4 as compared to tablet LT4, as patients had difficulty remembering and sticking to the treatment plan; a factor not taken in consideration as it is not yet available in Pakistan. A lower adherence rate was reported by a study in Lebanon, with significant improvements in adherence with good doctor-patient communication and regular appointments; as opposed to last minute appointment cancellations, patients with history of water pipes and alcohol intake per week [19]. A multinational, cross-sectional, internet-based study, conducted in 2016 [20], gathered data from pregnant women in 18 countries. The adherence rate was low in 17% while it was reported to be moderate and high in 44% and 39%, respectively. Younger age, unstable relationships and no folic acid intake were associated with lower adherence. In Hepp Z et al. [21], lower all-cause and hypothyroidism-related costs and resource utilization are reported in adherers as compared to non-adherers and a lower incidence of concurrent co-morbid diagnosis implying that compliance leads to both clinical and economical benefits in the long run.

From all data and literature, one common conclusion which can be drawn is, that healthcare professionals play a significant role in the management of hypothyroidism and can help reduce the barriers to optimal treatment by patient education about the drugs and their disease. Furthermore, involving pharmacists and nurses can help increase thyroid replacement in patients. Physicians should take into consideration the financial and social conditions, relationship status and mobility of a patient while prescribing medications. A keener emphasis on diet and addressing issues like sexual dysfunction (otherwise considered a taboo in Pakistani society), weight gain and lethargy in concerned patients helps build a better doctor-patient relationship and develops patient confidence in their healthcare provider.

## Conclusions

Hypothyroidism is a chronic progressive medical illness which demands strict compliance to medication for disease control. Like other chronic illnesses, patients with hypothyroidism also have difficulty in maintaining compliance to their treatment. Patients with hypothyroidism showed moderate adherence to their treatment. Patient education, regular endocrinologist visits, and affordability of medications increased medication compliance. Improving medication compliance demands a multi-faceted approach with robust efforts from both the health practitioner and also the patient. Health professionals should be willing to develop and implement potentially effective means to achieve this. 
